# Cellular Immunotherapy of Canine Cancer

**DOI:** 10.3390/vetsci5040100

**Published:** 2018-12-06

**Authors:** Selamawit Addissie, Hans Klingemann

**Affiliations:** Nantkwest Inc., Culver City, CA 99232, USA; Selamawit.Addissie@NantKwest.com

**Keywords:** canine immunotherapy, lymphocytes, natural killer cells, chimeric antigen receptors

## Abstract

Infusions with immune cells, such as lymphocytes or natural killer (NK) cells, represent one of several modalities of immunotherapy. In human patients with advanced B-cell leukemia or lymphoma, infusions with chimeric antigen receptor (CAR) T-lymphocytes have shown promising responses. However, the scientific and clinical development of cell-based therapies for dogs, who get cancer of similar types as humans, is lagging behind. One reason is that immune cells and their functionality in dogs are less well characterized, largely due a lack of canine-specific reagents to detect surface markers, and specific cytokines to isolate and expand their immune cells. This review summarizes the current status of canine cancer immunotherapies, with focus on autologous and allogeneic T-lymphocytes, as well as NK cells, and discusses potential initiatives that would allow therapies with canine immune cells to “catch up” with the advances in humans.

## 1. Introduction

After many years of trial and error, there is finally some success to report from human medicine, that the immune system can control, and even reverse, the growth of cancer. Unleashing immune cells (mostly lymphocytes) with monoclonal antibodies (mAbs) against checkpoint inhibitors, such as PD-1, PD-L1, and CTLA-4, have resulted in tumor regression/control in about 20%–30% of patients with various advanced cancers, in particular, melanoma, lung, or bladder cancer [1]. Further, infusion of autologous T-lymphocytes, engineered to express a chimeric antigen receptor (CAR) for the CD19 B-cell antigen, can induce remissions in patients with advanced lymphoblastic leukemia and lymphoma (reviewed in [2]).

Those emerging success stories in human medicine have spurred interest in developing cellular therapies for dogs with cancer [3]. Canine and human cancers bear similarities in terms of tumor localization, metastatic pattern, and response to treatment [4]. However, to develop effective immunotherapies for dogs, we need to learn more about the cells of their immune system, their characteristics, functionality, and interaction with other cells in the tumor environment. Currently, we know little about the different T-lymphocyte populations in dogs, and natural killer (NK) cells remain poorly defined. The innate immune “landscape”, and the impact of the tumor microenvironment, is largely unknown in dogs, whereas it is heavily researched in human medicine, as its relevance is increasingly recognized [5]. Since only a minority of tumor specific antigens have been described in dog tumors, specific and—compared to humans—effective mAbs, have not been developed, although a new mAb against CD20 by Elanco is getting some attention [6]. The therapeutic use of immune cells, obtained from the dog, enriched, activated, and possibly engineered, is just beginning to get some initial attention (reviewed in [7]).

It should not come as a surprise, though, that canine cancer is responsive to cell-based immunotherapies. Some 50 years ago, the Fred Hutchinson Cancer Center in Seattle pioneered bone marrow transplantation for dogs with advanced lymphoma [8]. Initially, it was thought that the curative effect of this procedure was exclusively due to the high doses of chemotherapy and radiation administered in preparation for the transplant. However, a larger retrospective data analysis confirmed that the relapse rate was lower in dogs who had received an allogeneic transplant from a littermate, compared to the infusion of the dog’s own (autologous) bone marrow stem cells, despite the same doses of chemotherapy and radiation to prepare for the graft. This “*graft versus leukemia/tumor effect*”—as it has become known—is largely mediated through allogeneic activated T-lymphocytes recognizing and responding to antigen differences, thereby also attacking any residual cancer cells [9]. By contrast, autologous lymphocytes, unless modified/activated, will not sufficiently recognize the malignant cells.

In addition to the fact that the patient’s own (autologous) lymphocytes do not “see” the cancer as foreign, it is known that the cancer can produce a host of molecules and factors that are inhibitory to the patient’s immune cells [10]. For example, it has been shown that every tumor releases tiny microvesicles or exosomes that contain molecules that can significantly affect the activity of immune cells [11,12]. Unless fresh, activated, and ideally engineered immune cells are infused, treatment with autologous blood, or even tumor infiltrating lymphocytes (TIL), will have limited efficacy, like in humans [13].

Autologous T-lymphocyte cells can become more effective killer cells when they are re-engineered with chimeric antigen receptors (CARs) that specifically recognize tumor-associated surface antigens [2]. However, CAR-T cells work best against hematological tumors, where they can “reach” the tumor site and the malignant cells. Solid cancers are trapped in a microenvironment that is characterized by increased fibrosis and irregular vascularization, leading to hypoxic areas and metabolic byproducts that can negatively affect immune cells [14]. In addition, a host of immune-suppressive cells, such as myeloid-derived suppressor cells (MDSC) and T-regulatory cells, as well as molecules such as TGF-beta or prostaglandins, can induce fibrosis and affect cytotoxic lymphocyte function [15].

## 2. Clinical Studies of Autologous Lymphocyte Infusions

Lymphocyte collections from dogs require leukapheresis, that usually takes a few hours, but is generally tolerated by the dog, with some sedation. The lymphocytes are then expanded on an engineered cellular feeder layer with additional cytokines (such as human IL-2) before being infused into the canine patient [16]. The challenge is to expand sufficient numbers of lymphocytes for treatment. Realizing this shortcoming, the group at MD Anderson Cancer Center developed an expansion protocol for dog lymphocytes that uses a feeder layer of human K562 cells, transduced with lentivirus to co-express human CD19, CD64, CD86, CD137L, and membrane-bound human IL-15 [16]. The K562 feeder cells are irradiated with 100 cGy, and loaded with anti-human CD3 mAb. Canine blood mononuclear cells are placed on this highly engineered feeder layer, and the canine T-lymphocytes are expanded in the presence of a high dose of (human) cytokines IL-2 and IL-21.

This expansion approach yields about 10 million CD3-positive T-lymphocytes, which are then infused into dogs with lymphoma who are pre-treated with a 19 week chemotherapy regimen [16]. The outcome, compared to historical controls, suggested a survival benefit for the group of dogs that had received the expanded lymphocytes. Although the ex vivo expansion protocol boosted the number of CD8-positive cytotoxic T-cells, those were not tumor specific. The study confirmed, though, that it is safe to infuse ex vivo activated autologous lymphocytes, and that recombinant human cytokines (IL-2, IL-21) can support the ex vivo expansion of canine T-cells, although relatively high doses are required. Since only historical data served as a control, it is difficult to conclude what the therapeutic benefit of this rather labor-intensive treatment is and, unfortunately, no follow-up studies were reported.

A few biotech companies have entered the field of autologous T-cell therapy for dogs. *Aurelius BioTherapeutics* (www.AureliusBio.com) collects 20 mL of blood from dogs with cancer, from which the lymphocytes are expanded over 2–3 weeks. It has not been disclosed how the lymphocytes are activated and expanded, and whether there is any benefit of this approach on tumor control. The company charges dog owners $6500 for this treatment. Further outcome data are eagerly awaited.

In order to make T-cell therapy treatment more cancer-cell specific, *Elias Animal Health* (www.Eliasanimalhealth.com) has added a vaccination step prior to lymphocyte collection [17]. The company prepares a vaccine from the excised tumor material that is then given intradermally to the dog several weeks before lymphocyte collection. The collected lymphocytes are expanded in the presence of human IL-2, and infused back into the patient, usually a couple of weeks later. Prior to the infusion, the dog may receive a brief cycle of chemotherapy which, in humans, has resulted in better acceptance of the lymphocyte treatment. Preliminary observations suggest that overall survival may be extended with this form of adoptive immunotherapy, suggesting that the immune intervention triggered a vaccine-like effect—patients live longer, even with the disease/cancer not completely gone. After all, turning cancer into a chronic disease with good quality of life would not be a such a bad outcome. The company is seeking regulatory approval, which would make it the first approved/commercial cell therapy for dogs.

Another type of autologous lymphocyte infusion, which has only been explored in the human setting, so far, utilizes tumor infiltrating lymphocytes (TIL) that are believed to be tumor-specific, as they are isolated from the tumor site, and expanded ex vivo to larger numbers [18]. The rationale is that TIL cells could recognize specific neo-antigens (neo-epitopes) on cancer cells that are not present on normal cells [19]. To be efficacious, this approach requires tumor excision and isolation of the infiltrating lymphocytes. Genetic mapping (DNA and RNA sequencing) of the tumor will identify mutations that are recognized by the TIL. Those few cells are then expanded ex vivo, and infused back into the patient. This has recently been described in a human patient with metastatic breast cancer who also received a checkpoint inhibitor [20]. If applicable for dogs, this (experimental) treatment would require deep knowledge of the canine genome and the mutations that are responsible for cancer. 

Recently, *chimeric antigen receptor (CAR)*-engineered T-cells have made major news in human immunotherapy [2,21]. CAR engineering involves “fusing” the antigen recognizing (Fab) part of a mAb into the T-lymphocyte membrane, such that the cytotoxic lymphocytes recognize a specific target and get activated upon binding to the cancer cell.

Remissions with CD19 recognizing CAR T-cells can be achieved in some 80% of human patients with advanced leukemia or lymphoma at the 4-week assessment point [2,21]. Relapses continue to occur, even within the first 6 months, and CAR T-cell treatment can also have significant toxicities, such as cytokine release syndrome (CRS), encephalopathy, and bone marrow suppression was reported in about one-third of treated patients [22]. Recently, a case of treatment-related leukemia was reported: the lentivirus that is used to transfer the CAR gene into lymphocytes was inadvertently inserted into a residual leukemia cell, promoting its transformation [23]. 

Some investigators have started to explore CAR-T cell therapy for dogs [24,25,26]. A team at MD Anderson Cancer Center transfected canine lymphocytes with a *human* HER-2 CAR, and showed that the human CAR can recognize and kill HER-2-positive canine osteosarcoma cell lines in vitro [24]. They also confirmed that HER-2-CAR specific T-lymphocytes from dogs can be successfully expanded ex vivo. However, the process is labor intensive, requiring an irradiated (100 cGy) feeder layer of human K562 cells on which the canine PBMC are layered with a mitogen, and human IL-21 is added to the culture. Those expanding T-cells are re-stimulated on day 7 of culture with IL-2, and fresh media fed every 3–4 days. No canine patients have been treated yet.

CAR-T expansion protocols for canine CD20 CAR were recently published [25,26]. T-cells from dogs were expanded on a layer of artificial antigen-presenting cells that had been engineered to express human CD32 and canine CD86 [25]. The T-cells added to the feeder layer were then stimulated with a canine CD3 monoclonal antibody, and further expanded with human cytokines (IL-2 and IL-21).

To transfect the CD20 CAR into the expanded T-cells, the investigators used electroporation of CAR mRNA [26]. Although this avoids the use of retro- or lentivirus, mRNA transfection has variable efficiency and, also, is only temporary, with degradation of the mRNA and loss of transcriptional activity within 24 to 48 h. One dog with lymphoma was treated with engineered T-cells, but had only a partial and transient response. This is not surprising, since we know from studies in humans that durable responses only occur when the infused CAR T-cells can expand in vivo [2,21]. The investigators also did not administer lymphocyte-depleting chemotherapy to the dog prior to infusion, which is quite common in the human treatment scheme, to eliminate suppressor immune cells and, in the case of dogs, reduce the risk of a canine anti-mouse antibody immune response. Pursuing non-viral transfection modalities is relevant, as any virally altered and infused cell products will require stringent containment and follow-up conditions for the canine patient.

The studies published, so far, confirmed that it is possible to generate CAR-T cells from dog lymphocytes, although the logistics and the costs are likely to be substantial [24,25,26]. They also pointed to another problem with immunotherapy for dogs: that it needs human cytokines to manipulate the T-cells, and that there are not sufficient immunological reagents available that are specific for dogs. Such reagents would allow for analysis of what is occurring at the cellular level, including which cytokines are produced. Human cytokines, often used to expand canine lymphocytes or NK cells, can be immunogenic. On the bright side, the study by Mata et al. [25] confirmed that some human CARs can cross-react with canine tumor antigens (as in the case of HER-2). Scientists at University of Pennsylvania are engineering human T-cells as a possible source for CAR-T cells for dogs, by using CRISPR technology to eliminate the HLA antigens and the T-cell receptor [27]. Unfortunately, the lack of HLA antigens can trigger the dog NK cells, whose function it is to recognize “non-self”, to attack the CAR-T cells.

## 3. NK Cells

In addition to T-lymphocytes, NK cells are increasingly being considered in humans as cellular therapeutics for cancer [28]. Human NK cells can be identified and separated from T-cells by the expression of CD56 and the lack of T-cell antigens, such as CD3 and the T-cell receptor (TCR). Canine NK cells do not express CD56—confusingly, they seem to express the T-lymphocyte marker CD5, although at low level [29]. To isolate NK cells from canine PBMC, either CD3 or CD5 depletion can be used, that allows for enrichment of a non-T- and non-B-cell population [29]. Recently, an antibody against the canine NKp46 (NCR1) receptor has been described [30], that may help in NK cell characterization and isolation—unfortunately, it is not yet commercially available. Based on those markers though, the relative percentage of NK cells in canine PBMC is only about 5% of all lymphocytes, compared to about 10–15% in humans.

Only one clinical trial using NK cells in dogs has been reported so far [31]. The group at University of California Davis isolated NK cells from blood lymphocytes based on CD3 negativity, and CD5 low surface antigen expression. To expand the number of NK cells, the investigators used the human K562 cells as a feeder layer, which were engineered to express human IL-21. In a preclinical subcutaneous mouse model, one group received focal radiation to the tumor site, while the other group did not. NK cells were given intravenously. The data suggested a benefit (tumor shrinkage and improved survival) for the mice receiving the NK-cells after radiation. Biopsies obtained from the tumor site showed that more NK cells had been retained at the tumor site in the radiation group [31]. The investigators subsequently treated 10 dogs with locally advanced, non-metastatic osteosarcoma, with radiation followed by intra-tumor NK cell injection. Dogs also received 250,000 IU/kg of human IL-2. There was tumor shrinkage and 5/10 patents had no pulmonary metastasis at the 6-month endpoint. Considering the limitations of this study, it may still be possible to conclude that pre-treatment radiation could provide an additional antitumor effect for cellular immunotherapy. It is known that radiation induces adhesion molecules, such as ICAM-1, on tumor cells, which bind to the LFA-1 on NK cells [32]. Moreover, radiation induces expression of “stress ligands” on tumor cells, which become targets for the activating NKG2D receptor on NK cells [33].

In addition to providing “spontaneous” cytotoxicity (in contrast to T-cells, NK cells do not require any antigen priming), NK cells are also important effector cells for monoclonal antibody (mAb)-mediated antibody-dependent cell-mediated cytotoxicity (ADCC), a mechanism that, in humans, accounts for most of the antitumor effect of IgG1 and IgG3 mAbs [34]. While the Fc-receptors are quite well defined in humans, they are less well characterized in dogs, which may account for the limited efficacy of mAb infusions [35]. Efforts are underway to generate “canine-ized” mAbs to prevent anti-human IgG antibody formation [36].

## 4. Clinical Studies with Xenogeneic Lymphocytes

In the mid-nineties, the group at Wistar Institute infused cells from a continuously growing human NK/T cell line (TALL-104) into dogs [37,38,39]. It was an unusual approach, as the cells were not only xenogeneic, but they also had been isolated from a human patient with leukemia. To determine the safety and potential efficacy of TALL-104 infusions in dogs, a phase I trial in pet dogs with spontaneously occurring refractory tumors was conducted [37]. Due to the origin of the TALL-104, from a patient with a malignant disease, the cells were lethally irradiated (40 Gy). Based on in vitro data, some canine patients received OKT3 mAb and cyclosporine to prevent alloimmunization.

Although all dogs developed anti-human antibodies after about two weeks of TALL-104 treatment, various degrees of clinical responses were observed: one dog achieved a complete remission, and two dogs showed a partial response and stable disease. Importantly, no grade 3 or 4 side effects were seen. Noteworthy are the clinical responses seen in dogs with malignant histiocytosis, an aggressive and universally fatal disease in canines [38]. In addition to the one dog that achieved a complete response in the phase I trial, three other dogs with this tumor showed no evidence of disease for a much longer time than one would expect in the course of this illness. TALL-104 cells were also given to several dogs with lymphoma and malignant mammary tumors [39]. All of the pets remained disease-free 6–18 months after cell therapy, despite their poor prognostic factors. However, because of some difficulties in expanding TALL-104, this cellular therapy was not pursued any further.

**Figure 1:**
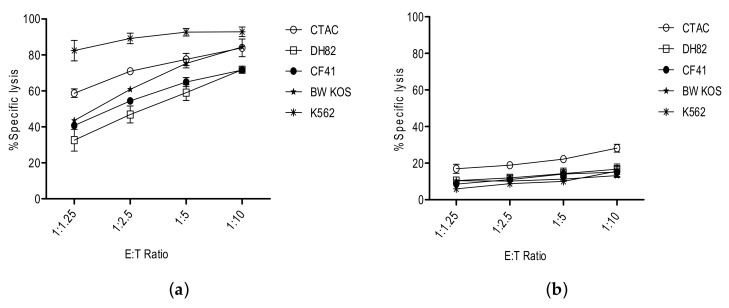
Comparison of tumor cell lysis by NK-92 (**a**) and primary canine NK cells (**b**).

We have tested a spectrum of canine cancer cell lines as to whether they can be killed by the *human* natural killer cell line NK-92 [40,41]. Figure 1 shows that NK-92 killed all tested canine cancer targets very effectively. Not surprisingly, fresh, non-activated dog NK cells did not kill the same targets to a meaningful degree. It is known that blood-derived NK cells need some form of activation by cytokines (IL-2, IL-15, or IL-21) [42].

Cytotoxicity of NK-92 and canine NK cells was tested against various canine cancer cell lines: DH82 (histiocytosis), CF41 (mammary tumor), and BW KOS (osteosarcoma). The human K562 and the canine CTAC (thyroid adenocarcinoma), which are known to be sensitive to NK killing, were used as human and canine positive control cell lines, respectively. Canine NK cells were obtained from the peripheral blood of healthy dogs by Ficoll-Hypaque density gradient centrifugation, followed by depletion of CD5-positive cells to remove the T-cell population. The enriched canine NK cells were used fresh, without cytokine stimulation and expansion. Different effector to target ratios are presented for the 4 h flow cytometric-based cytotoxicity assay.

## 5. Where to Go from Here?

It has become clear that the tumor microenvironment plays a major role as to how immune cells (canine or human) can recognize and react with cancer cells. In addition to a potentially distorted blood flow that creates a hypoxic, immunosuppressive environment, tumor-resident cells, such as T-regulatory cells and myeloid-derived suppressor cells (MDCS), as well as an array of tumor-derived products, can negatively impact immune cells [43]. Further immunotherapeutic approaches in dogs should address the suppressive factors in the tumor microenvironment.

Chemotherapy and radiation—if used metronomically and at low doses—can induce *immunogenic* cell death, as opposed to high doses that causes *tolerogenic* death [44]. Higher doses of chemotherapy also paralyze patient’s immune cells, and induce escape mutations. Metronomic chemotherapy has been used for dogs before, with minimal side effect and potential benefit [45]. Unfortunately, these studies were not done in combination with immunotherapy, particularly infusion of immune competent lymphocytes or NK cells. “Taking the brakes off” the patient’s own lymphocytes, with checkpoint inhibitors, remains a goal, but, so far, no such therapeutics—with one exception [46]—have been developed for dogs.

Intra-tumor injection of NK cells can induce tumor regression and lasting responses [31]. It is known from studies in mice that intratumor injection of NK cells can mediate a vaccine effect [47]. Such a “local” immunotherapy approach should be less expensive, since fewer cells are used for direct injection. Modulation of this response with local tumor irradiation, metronomic chemotherapy, and factors that positively regulate the tumor microenvironment, could provide lasting responses, at a cost that dog owners will be comfortable with.
